# The importance of the nutritive value of old bones in the diet of Bearded vultures *Gypaetus barbatus*

**DOI:** 10.1038/s41598-017-08812-2

**Published:** 2017-08-14

**Authors:** Antoni Margalida, Daniel Villalba

**Affiliations:** 1 Department of Animal Science, Faculty of Life Sciences and Engineering, University of Lleida, 25198 Lleida, Spain; 2 Division of Conservation Biology, Institute of Ecology and Evolution, University of Bern, CH-3012 Bern, Switzerland

## Abstract

Vultures are central-place foragers and need to optimize their foraging behaviour to offset travel costs by increasing their energy gain. This process is more obvious in certain vulture species that do not feed their young by regurgitation and so must carry food items back to the nest. The Bearded Vulture *Gypaetus barbatus* is the only species with a bone-diet based. We analysed the chemical composition of bones and the age-related changes in their nutritive value to assess the differences in energy content between bones of differing age, body part and species. We found differences between specific anatomical parts, species and the age of the bones. Fresh bones contain 108% as much energy as fresh meat and, interestingly, dry bones retain 90% of the protein found in fresh bones. Dry femurs weighing 140 g retain enough protein to be comparable to 111 g of fresh meat, in energy terms. Compared to meat-eating species, the specialized osteophagous diet of the Bearded Vulture seems to have certain advantages. A better understanding of nutrient levels in food remains could help to improve theoretical foraging models, assist in conservation management, and even improve our understanding of the use of bones by early hominids.

## Introduction

Central-place foragers are those that gather food in their surroundings and then carry it back to a home base, such as a nest, for consumption, storage or feeding to their young. They transport food items rather than consume them at the place of discovery and are therefore sensitive to the costs involved in carrying food, which are consequently kept as low as possible^1^. Their foraging behaviour therefore tends to focus on the selection of food items rich in important nutrients as a means of redressing cost/benefit imbalances and optimizing the results of foraging^2^.

As the distance travelled to find food increases, central-place foragers should compensate for the increased travel costs by increasing the energy gain from the food collected^3, 4^. An example is the case of certain obligate scavengers, whose diet is based on carrion which remains useably fresh for only a limited period of time and that do not feed their young by regurgitation. How variation in the nutritional quality of food over time affects food choice is therefore, key to improving theoretical models of optimal foraging for such species. Better analysis of this issue will not only help in understanding the evolutionary ecology of central-place foraging animals, but the foraging behaviour of early hominids as well^5–7^.

The Bearded Vulture *Gypaetus barbatus* inhabits mountainous regions in Eurasia and Africa where low environmental temperatures lead to an increase in basal metabolic rate and energy expenditure. Its diet mainly comprises the bone remains of medium-sized ungulate carcasses^8, 9^ and in most cases requires the use of bone-breaking sites, or ossuaries, to fragment bones for consumption. Rocky surfaces provide ossuaries onto which bones are deliberately dropped from a height by Bearded Vultures in flight, the bone remains breaking up as a result^10, 11^. The spatio-temporally sporadic distribution of ungulate carcasses results in unpredictable costs in time and effort invested in searching for bone remains. Although bones have an apparently negligible nutritional content, it has been calculated that for every 100 g of bone ingested, Bearded Vultures can actually absorb 387 kJ (compared to 440 kJ from the same weight of meat), demonstrating that a bone-based diet is energetically almost as rich as a meat-based diet, due to the high fat content of bones^12, 13^. In addition, compared to a meat-based diet, a bone-based diet has the important advantage that bones remain edible for much longer periods than do soft tissues^13^. This enables Bearded Vultures to effectively store bones at ossuaries, perches and nests^10, 11^.

As central-place foragers, processing bones before carrying them to their young benefits Bearded Vultures by reducing the weight of food items and hence the energy required to carry them to the nest^14, 15^. They select the most nutritive, fatty parts of carcasses (those with a high percentage of oleic acid) regardless of bone size^15^. This maximises foraging effort by optimising foraging time in relation to the amount of energy gained from the food. However, the longevity of the nutritive value of food items may limit the effectiveness of this type of food selection. The impact of this factor has been underestimated and is fundamental to understanding the particular niche of this specialized species.

This study set out to analyse the effect of bone age on the nutritional value of carcass bones and to assess the energy provided by bones from different anatomical regions of different ungulate species. Knowing how bones retain their nutritive value over time has interesting implications for understanding Bearded Vulture ecology, will improve theoretical foraging models and the management and conservation of this threatened species, and also provides tools to enable a better understanding of early hominid evolution.

## Results

The chemical composition of the sampled bones differed between species, bone age and anatomical part (Table 1). Sheep bones contained less dry matter (F = 143.9, df = 1, p < 0.0001) and fat (F = 428.3, df = 1, p < 0.0001) but higher ash (F = 436.0, df = 1, p < 0.0001) and protein (F = 34.1, df = 1, p = 0.0006) than those of pigs. As expected, fresh bones contained less dry matter than dry bones collected in the field. Fresh bones contained less ash (F = 2091.3, df = 1, p < 0.0001) but more protein (F = 53.2, df = 1, p < 0.0001) and, especially, more fat than dry bones (F = 1944.3, df = 1, p < 0.0001). The two long bones contained similar levels of dry matter and protein, although femurs had more fat and less ash than tibias. Scapulas contained less dry matter, ash and fat, but more protein, than long bones.

**Table 1:** Chemical composition of bone according to species, preservation time and anatomical part (Mean ± s.e.).

Effect^1^	Dry matter (%)	Chemical composition of bone (g/100 g DM)		
		Ash	Fat	Protein
**Species**				
Sheep	74.77^b^ ± 0.84	62.76^a^ ± 0.41	5.06^b^ ± 0.36	30.85^a^ ± 0.39
Pig	79.66^a^ ± 0.84	54.24^b^ ± 0.41	13.51^a^ ± 0.36	28.46^b^ ± 0.39
**Age of the bones**				
Fresh	60.92^b^ ± 0.84	49.17^b^ ± 0.41	18.28^a^ ± 0.36	31.14^a^ ± 0.39
Dry (>3 months)	93.51^a^ ± 0.84	67.84^a^ ± 0.41	0.28^b^ ± 0.36	28.17^b^ ± 0.39
**Anatomical part**				
Tibia	80.58^a^ ± 1.02	60.50^a^ ± 0.51	9.52^c^ ± 0.44	27.85^b^ ± 0.47
Femur	76.77^a^ ± 1.02	58.22^c^ ± 0.51	11.53^a^ ± 0.44	27.42^b^ ± 0.47
Scapula	74.29^b^ ± 1.02	56.79^b^ ± 0.51	6.79^b^ ± 0.44	33.70^a^ ± 0.47

^1^Species and Anatomical part effect calculated using fresh and dry bones. Age of the bones effect calculated using both species. Different superscripts indicate significant differences between values (p < 0.05).

Considering only the differences in fat and protein content (Table 1), and assuming energetic values of 16.7 kJ/g for fat and 37.6 kJ/g for protein, the estimated energetic content per 100 g of raw bone varied: pig bones provided 782 kJ, whereas sheep bones had only 527 kJ (33% less); fresh bones provided 63% more energy than dry bones; tibias and femurs had similar energy contents (664 kJ and 685, respectively); and scapulas contained the least energy (608 kJ, 12% less than long bones).

A statistically significant interaction for age*anatomical part was detected in the dry matter content (F = 9.6, df = 2, p = 0.0024). Although dry bones from all anatomical parts had equal amounts of dry matter, differences in dry matter were detected between anatomical parts in fresh bones. The interaction between age*species also showed statistically significant differences in ash content (F = 64.0, df = 1, p < 0.0001). The difference in ash content between fresh and dry bones was greater in pigs than in sheep (Table 1).

**Figure 1:**
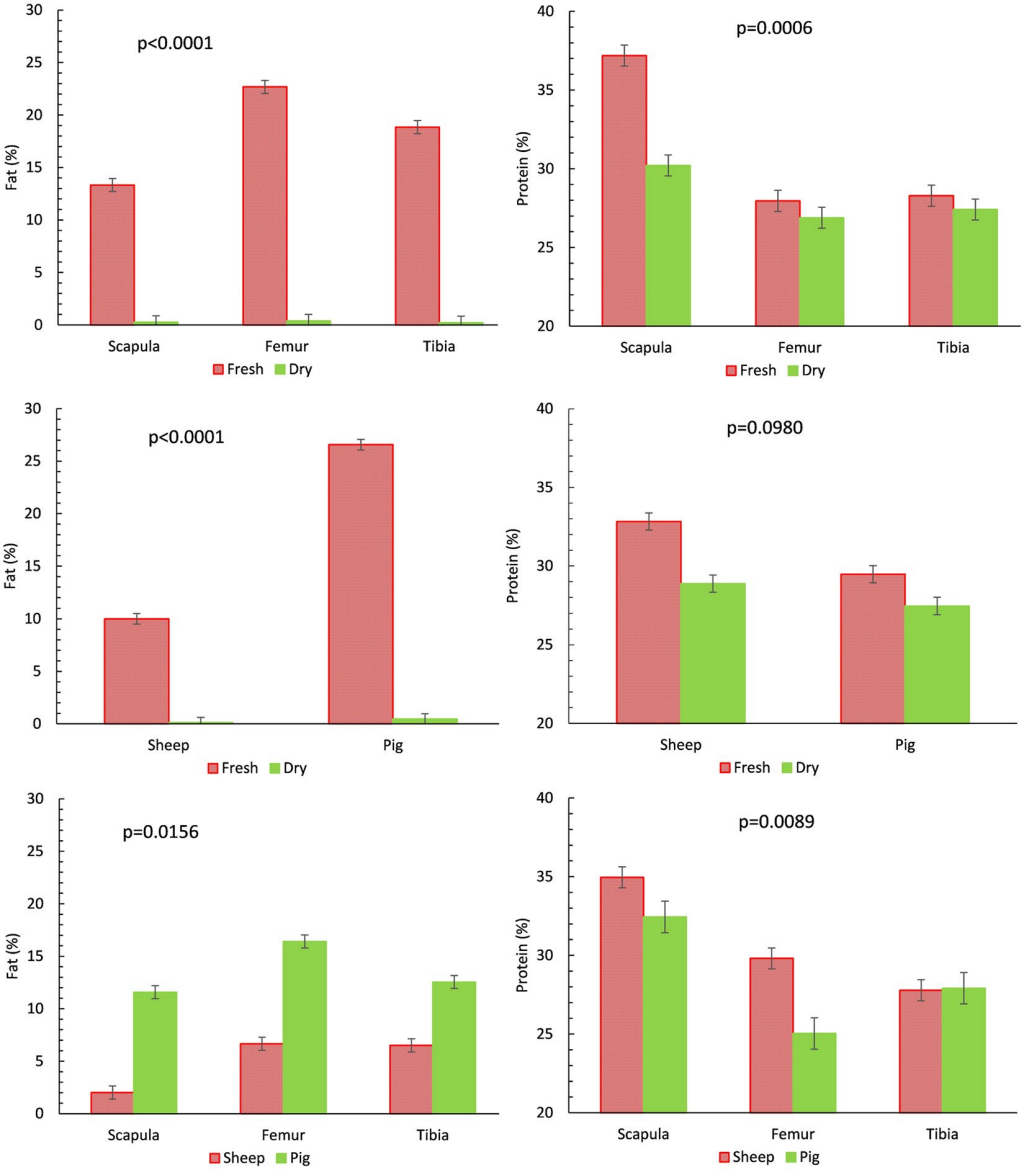
Fat and protein content for the interactions between anatomical part (scapula, femur and tibia), species (pig vs sheep), and age of the bones (fresh vs dry [>3months]). Bars represent ±s.e. P-values denote the significance of the interaction in the model.

The interactions age*anatomical part and species*anatomical part were significant for fat and protein content (Fig. 1). Fat content was also affected by the interaction age*species. Increasing bone age clearly reduced the fat content of bones, although values between species and anatomical parts did not differ significantly (Fig. 1). On the other hand, fresh bones showed fat content differences by both species and anatomical part. Protein content varied less between fresh and dry bones, although scapulas (which had the highest protein level when fresh) lost more protein when dry, in contrast to long bones which maintained their protein levels with increasing age.

Protein and fat levels were highest in fresh bones. We estimated the energy content of bones by assuming an energetic value based on the energy content of protein and fat. A standard fresh sheep femur (average weight 220 g) provides Bearded Vultures with 1055 kJ, equivalent to 239 g of fresh mutton (514 kJ/100 g)^16^. Dry sheep femurs weighing 140 g that have lost fat and water still contain as much protein as 111 g of fresh meat in energy terms.

## Discussion

Fresh rib bones have a mean water content of 32%; and a bone dry weight comprising 54% mineral and 46% organic content^13^. Due to their high fat content, mammal bones reportedly have a greater energy content than muscle tissue (6.7 vs 5.8 kJ/g respectively)^8, 12^. Our study revealed differences in fat and protein content between different anatomical parts (femurs have more fat and scapulas more protein), species (pig bones have twice as much fat as sheep bones), and bone age (dry bones contain practically no fat but maintain their protein levels). The variation between the composition of bones from different anatomical parts and species (in domestic as well as wild ungulates) have previously been studied using oleic acid as a proxy for nutritive content^17^ without taking temporal effects into account (i.e. the loss of nutritional value over time). Our results also show that, based on Williams’ values of mean energy of different cuts of mutton meat (514 kJ/100 g)^16^, that fresh bones contain 108% of the energy provided by fresh meat. Interestingly, dry bones retain 90% of their dry matter protein compared with fresh bones. Fat content (probably corresponding to the fat in bone marrow) is lost as bones dry in the open air which reduces their energetic value (authors unpub. data). However, from an energetic point of view, although a fresh long bone can deliver almost the same amount of energy as fresh meat weight for weight, this calculation is based on the gross energy delivered by protein and fat, and this issue should be understood in light of the special digestive abilities of Bearded Vultures. Bone is a dense food and it has been estimated that it takes 24 hr to digest^13^. The Bearded Vulture’s stomach wall contains a high density of acid-secreting cells, and probably creates a highly acidic environment^13^. The pH levels in Bearded Vultures’ stomachs are unknown, but could be less than 1 judging by the stomach pH of 0.7 reported for *Gyps africanus* ^13^. Stomach pH levels of 1.6 (Falconiformes) and 2.4 (Strigiformes) have also been reported in other raptor groups whose diet includes bones^18^. The digestion, absorption and assimilation of bones with various protein and fat contents (i.e. fresh or dry) under these pH levels deserves further study in order to better understand the amounts of energy and nutrient that bones can actually provide as a food source.

Some studies on the Pyrenean Bearded Vulture conclude that food availability has no quantitative effect on population trends, and is not a limiting factor in this species^19, 20^. Nevertheless, taking account of qualitative data on the anatomical and temporal variation in the nutritive contents of bone remains could improve these models and lead to better management practices (e.g. provision of the most beneficial types of bone at supplementary feeding sites). For example, assuming: a) a daily energetic requirement of 1687.3 kJ (the average of 1570.05 kJ at 30 °C and 1804.51 kJ at 0 °C, according to the basal metabolic rate of Bearded Vultures measured by^21^; and b) that a fresh femur has an energetic content of 1055 kJ vs 671.4 kJ for a dry femur, we can estimate that an individual vulture requires 583 fresh femurs to provide its annual energetic requirements compared with 917 if the femurs are dry. While this is a conservative estimate, assuming that the assimilation efficiency of vultures on a meat-diet is 0.86^22^, it does reflect the lower nutritive value of old bones (>3 months)–about half that of fresh bones, probably due to the progressive loss of marrow content. Additional variations in the nutritive value of bones probably occur seasonally, bones probably retaining their fat content for longer during colder periods of the year. Food availability models should therefore incorporate more detailed estimates of bone nutritive value and allow for seasonal differences as well as differences due to bone age and body part. The time component is of particular importance as marrow is lost as bones age, resulting in significantly lower nutritive value as time goes by. Considering the importance of differences in the nutritive value of different anatomical parts, studying which types of bone are preferred can help to improve estimates of actual food availability. For example, mandibles, scapulas, or skins with lower oleic acid content^15^ provide negligible food value in the diet of Bearded Vultures, and as a result are not suitable for provision at supplementary feeding sites. The management and conservation impact of supplementary feeding sites can be enhanced by regular provision of the most nutritive remains (femurs, tibias) to maximise food quality.

Although our results are based on analyses of domestic livestock species, they can probably be extrapolated to wild ungulates of similar size and characteristics^23^. Bones remain edible for much longer than meat, which decays and goes rotten due to bacterial action, giving specialized osteophagous species such as the Bearded Vulture an advantage compared to meat-eating species. Bearded Vultures utilize ossuaries, nests and perching sites to store food, and recover bones when food is scarce or when adverse weather conditions make flights in search of food difficult^10^. Indeed, it has been suggested that Bearded Vultures might even prefer to eat old bones than fresh ones because they will have lost about 30% of their weight and are hence easier to carry^8, 13^. As our results show, dry bones that have lost fat and water contain increased ash percentages as a result, but still contain sufficient protein to be comparable to fresh meat in energy terms. Hence, this specialized vulture can utilize old remains to carry them through periods of food shortage or survive in lower quality habitats where food supplies are limited.

Our results also have relevance to studies on optimal foraging of humans in various settings. Bone marrow has long been recognised as a valuable source of energy, and its fat and nutrients were exploited by prehistoric and historic peoples in many different environments^24, 25^. The contribution of optimal foraging studies of humans to the development of foraging theory has been noted by^24^.

Early hominids may have used avian scavengers as a means of locating carrion as a food resource^26, 27^. The rapid deterioration of the nutritive value of meat–due to bacterial contamination and decay–could be compensated for by the extra time over which bones remain edible^28^.

## Material and Methods

During 2013 we collected fresh and dry bones from domestic pigs *Sus scrofa* var. dom and sheep *Ovis aries*. All of the sampled individuals came from the same study area (Lleida, NE Spain). Fresh sheep and pig bones were collected from slaughterhouses and then frozen until processing. In addition, dry sheep and pig bones that had been left in the open air for > 3 months (range 3–12 months) were obtained from Bearded Vulture supplementary feeding sites in the wild. Four different animals of each species (sheep and pig) were sampled, and three bone types examined (fresh and dry); two long bones (tibia and femur) and one large flat bone (scapula) (see Table S1, Supplementary Information).

Sample bones were skinned and the meat removed, and then dried for more than 24 h at 60 °C to obtain the dry matter content (DM). The bones were then smashed into pieces about 3 cm long using a hammer, and then ground with cutting equipment (Thermomix, Vorwerk) until they could be passed through a 5 mm mesh sieve.

Chemical analyses of the ground-up samples were performed using two replicates per bone. The ash content was calculated after incineration at 700 °C until constant weight. The protein content was determined by sulphuric digestion and nitrogen distillation following the official AOAC method 2001.11, in which it is assumed that crude protein is equivalent to kjeldhal nitrogen multiplied by 6.25^29^. The fat content of the samples was determined by shoxlet extraction using petroleum ether without previous hydrolysis, following the official AOAC method 991.36^29^. The overall energy in each bone type was calculated using an energetic value based on the gross energy content of protein and fat.

The effects of animal species (Spec), anatomical part (Anat) and bone age (Age) on the chemical composition of bones (ash, protein and fat) were analysed using a linear model including the main effects (Spec, Anat and Age) and all of the interactions between them. A general linear model (GLM) procedure^30^ was used to obtain the significance levels of the main effects and interactions (data available at Table S1, Supplementary Information). The separation of the means was performed using a t-test. The level of significance was set at 5%.

## Electronic supplementary material

Supplementary Information
