# Supplementary material for: The importance of the nutritive value of old bones in the diet of Bearded vultures Gypaetus barbatus
Source: Sci Rep. 2017 Aug 14;7:8061. doi: 10.1038/s41598-017-08812-2 (PMC5556076; doi:10.1038/s41598-017-08812-2)
Supplement: Supplementary file 1 — Supplementary Information [file 41598_2017_8812_MOESM1_ESM.pdf]

Supplementary Information

The importance of the nutritive value of old bones in the diet of Bearded vultures *Gypaetus barbatus*

Antoni Margalida[1,2], Daniel Villalba[1]

[1] *Department of Animal Science, Faculty of Life Sciences and Engineering, University of Lleida, 25198 Lleida, Spain. Tel: (+34) 973 003 721. E-mail: amargalida@ca.udl.cat*

[2] *Division of Conservation Biology, Institute of Ecology and Evolution, University of Bern. CH-3012. Bern, Switzerland.*

Table S1. Chemical composition values of bone according to species, preservation time and anatomical part.

Sample id	Species	Preservation	Anatomical	Ashes	Fat	Protein	DM
22	Ovis aries	dry	femur	71.8788306	0.1108281	26.9844986	90.532
112	Ovis aries	fresh	femur	54.8159291	13.5108433	30.9319164	52.068
113	Ovis aries	fresh	tibia	57.8712388	12.2569111	28.5480856	66.175
114	Ovis aries	fresh	tibia	57.4303536	13.6600074	27.3457232	66.172
56	Sus scrofa var. dom	dry	scapula	63.6159666	0.33883038	29.29263	97.829
61	Sus scrofa var. dom	dry	femur	66.3501699	0.77604956	24.8367321	97.724
70	Sus scrofa var. dom	dry	tibia	67.43301	0.160305	28.2823173	98.214
122	Sus scrofa var. dom	fresh	femur	41.9264388	32.7466968	25.1219446	67.76
116	Sus scrofa var. dom	fresh	scapula	38.6681131	23.4533145	36.1104604	54.86
126	Sus scrofa var. dom	fresh	tibia	44.1685674	23.860375	28.7258791	70.99
121	Sus scrofa var. dom	fresh	femur	41.1537623	31.7034167	24.5288136	68.85
93	Ovis aries	dry	femur	68.2483977	0.24594404	30.0652878	91.83
115	Sus scrofa var. dom	fresh	scapula	43.6324493	22.0404344	33.7967002	57.34
127	Sus scrofa var. dom	fresh	tibia	45.8106108	25.5942575	28.5332751	66.62
67	Sus scrofa var. dom	dry	tibia	66.8346373	0.55951082	26.1169655	91.235
57	Sus scrofa var. dom	dry	scapula	64.0799015	0.46970201	30.5649895	92.199
60	Sus scrofa var. dom	dry	femur	67.1973131	0.41071424	25.6557159	92.365
87	Ovis aries	dry	scapula	66.867676	0.1449802	29.6752028	91.366
27	Ovis aries	dry	tibia	71.5480687	0.0625919	27.9369353	92.719
90	Ovis aries	dry	tibia	72.8876901	0.05016662	27.2934454	92.557

7	Ovis aries	dry	scapula	67.0962148	0.08292337	31.3038484	93.546
109	Ovis aries	fresh	scapula	53.8333819	4.11468775	40.9284909	53.036
110	Ovis aries	fresh	scapula	56.5236948	3.7177413	37.9180104	54.173
111	Ovis aries	fresh	femur	54.1666329	12.7692992	31.2532477	53.038
